# Parental mental health conditions and use of healthcare services in children the first year of life– a register-based, nationwide study

**DOI:** 10.1186/s12889-021-10625-y

**Published:** 2021-03-21

**Authors:** Signe Heuckendorff, Martin Nygård Johansen, Søren Paaske Johnsen, Charlotte Overgaard, Kirsten Fonager

**Affiliations:** 1 Department of Social Medicine, Aalborg University Hospital, Havrevangen 1, 9000 Aalborg, Denmark; 2 Danish Center for Clinical Health Services Research, Department of Clinical Medicine, Aalborg University & Aalborg University Hospital, Fredrik Bajers Vej 5, 9220 Aalborg East, Denmark; 3 Unit of Clinical Biostatistics, Forskningens Hus (Aalborg University Hospital Science and Innovation Center), Sdr. Skovvej 15, 9000 Aalborg, Denmark; 4 Public Health and Epidemiology Group, Department of Health Science and Technology, Aalborg University, Niels Jernes Vej 14, 1.sal, room 3-214, 9220 Aalborg East, Denmark; 5 Department of Clinical Medicine, Aalborg University, Forskningens Hus, Sdr. Skovvej 15, 9000 Aalborg, Denmark

**Keywords:** Parental mental health, Child health, Healthcare services, Mental health conditions

## Abstract

**Background:**

Parental mental health conditions have been associated with increased morbidity and use of healthcare services in offspring. Existing studies have not examined different severities of parental mental health conditions, and the impact of paternal mental health has been overlooked.

We examined the association between two severities of parental mental health conditions and use of healthcare services for children during the first year of life and explored the impact of both maternal and paternal mental health conditions.

**Methods:**

This register-based cohort study included all live-born children born in Denmark from 2000 to 2016. Information on socioeconomics, diagnoses, drug prescriptions, and healthcare contacts was extracted from nationwide public registries. Parents were grouped according to severity of mental condition based on the place of treatment of the mental health condition. Negative binominal regression analyses were performed to estimate the incidence rate ratio (IRR) of contacts to general practice (GP), out-of-hour medical service, emergency room (ER), and out- and inpatient hospital contacts during the first 12 months of the child’s life.

**Results:**

The analyses included 964,395 children. Twenty percent of the mothers and 12 % of the fathers were identified with mental health conditions. Paternal mental health conditions were independently associated with increased risk of infant healthcare contacts (GP IRR 1.05 (CI95% 1.04–1.06) and out-of-hour IRR 1.20 (CI95% 1.18–1.22)). Risks were higher for maternal mental health conditions (GP IRR 1.18 (CI95% 1.17–1.19) and out-of-hour IRR 1.39 (CI95% 1.37–1.41)). The risks were even higher if both parents were classified with a mental health condition (GP IRR 1.25 (CI95% 1.23–1.27) and out-of-hour contacts IRR 1.49 (CI95% 1.45–1.54)), including minor mental health condition (GP IRR 1.22 (CI95% 1.21–1.24) and out-of-hour IRR 1.37 (CI95% 1.34–1.41)). This pattern was the same for all types of healthcare contacts.

**Conclusions:**

Both maternal and paternal mental health conditions, including minor mental health conditions, were associated with increased utilization of healthcare services. Focus on both parents’ mental health conditions (even if minor) may be warranted in service planning.

**Supplementary Information:**

The online version contains supplementary material available at 10.1186/s12889-021-10625-y.

## Background

Poor parental mental health has been associated with a number of negative short- and long-term consequences for the health and well-being of a child. These consequences include the increased use of healthcare services [1–11]. Although it is generally acknowledged that both fathers and mothers influence their children’s health and lives [12–16], the father’s role has rarely been examined. In most studies of child healthcare, the father’s mental health are not included at all [1, 3, 4, 7, 8, 10, 11]. Other studies have analyze father’s mental health only as a control variable for which adjustments are made [2, 6, 17]. The number of adults in the household or adults linked to the child’s record are often considered without taking into account whether either the mother or father have depression [5, 9]. Thus, the effect of both father mental health and the combined effect of mother and father mental health has not been assessed.

Maternal depression based on either diagnoses or self-reported symptoms has been examined extensively and found to be positively associated with both hospitalization of the child [1, 4–8] and utilization of the emergency department [5, 6, 8, 9]. Moreover, children of mothers with depressive symptoms have been reported to visit general practice (GP) more [2, 3, 5, 6, 9, 10] and have been observed to have an increased risk of injury [11]. Maternal anxiety has also been studied and the findings have generally been comparable with the findings for depression in mothers [11]. Although mental health conditions such as depression include a wide range of symptoms, severities, and thus disabilities, existing literature does not distinguish between different severities of studied diagnoses or symptoms. As a result of this, the impact of different degrees of parental mental health conditions is unknown. This knowledge is needed to prioritize and plan services and interventions for both children and their parents. Since the GPs in Denmark are gatekeepers treating minor mental health conditions (such as minor depression or anxiety) and referring moderate to severe mental health conditions (such as severe depression, personality disorders or schizophrenia) to private psychiatrist or psychiatric hospital, the Danish registers provide the opportunity to differentiate between the severity of mental health conditions. As mental health conditions tend to be chronic and relapse, we included registered mental health conditions within 5 years prior to the birth of the child, thus also including mental health problems during pregnancy.

Many studies are limited by being cross-sectional [3–5, 7, 18, 19] which has led to concerns about the timing of exposure and outcome. Studies including self-reported symptoms [4, 7, 8] are limited by recall bias, and a register-based study provides control of exposure and outcome and ensures health professional evaluation of the mental health problems.

To address these gaps in existing knowledge, this register-based study aimed to examine the association between two severities of poor parental mental health and the child’s use of healthcare services during the first year of life while exploring the impact of both maternal and paternal mental health conditions.

## Methods

### Study design and data sources

We conducted a register-based cohort study using nationwide registers. Data linkages were achieved via the personal identity number (CPR number) which is assigned to all Danish residents at birth or upon receiving residency in Denmark [20]. Register keepers at Statistics Denmark carried out data collection and register linkage with all data being anonymized before the researchers gained access.

Information about parental diagnoses, birth-related variables of the child as well as both child in- and outpatient hospital contacts and emergency department (ER) contacts were obtained from The National Patient Register [21]. The diagnoses were encoded using the International Classification of Diseases, 10th Revision (ICD-10). The Danish Health Service Register for Primary Care [22] provided information on contacts to GPs as well as contacts to private psychiatrists and psychologists. Information on reimbursed drug prescriptions was obtained from the Danish National Prescription Registry which uses the Anatomical Therapeutic Chemical (ATC) Classification System [23]. Data on parity were accessed through the Danish Medical Birth Register [24] and on deaths through the Danish Death Register. Information on parental highest completed education was extracted from the Population Education Registry [25], and family income was obtained from the Income Statistics Register [26]. Date of birth, parental civil status, child gender and information on whether the parents lived both together and with the child was obtained from the Danish Civil Registration System [20]. The included Danish registers are generally considered to be high quality with complete long-term follow-up [27].

### Settings, study population and follow-up

The Danish healthcare system is characterized by free access. Services at general practice (GP) and public hospitals are funded by the Danish tax system and are free of charge. Additionally, acute medical assistance is only delivered by public services. GPs serves as gatekeeper for hospitals, and contacts to the hospitals require a referral from a GP. GPs are available during regular daytime hours. In case of illness outside of normal openings hours, an out-of-hour medical service is available.

All live-born children born in Denmark from January 1, 2000 to December 31, 2016 were identified and followed during the first 12 months of their lives. Children were excluded if they died, emigrated during the first year of life, or did not live with either biological parent after birth. Further, we excluded children of parents that did not live in Denmark at several time point during the exposure period since exposure information from outside Denmark were not available.

### Parental mental health

Parental mental health was categorized in three sub-categories (no mental health condition, minor and moderate-severe) using The National Patient Register, The Danish Health Service Register for Primary Care, and the Danish National Prescription Registry. In Denmark, minor mental health conditions are treated by GPs or psychologists in the primary healthcare sector and are thus not registered in The National Patient Register [28].

GPs serve as gatekeepers in the primary healthcare sector. These professionals refer more severe mental health conditions for assessment and treatment in the secondary healthcare sector (in- and outpatient wards at the hospitals or private psychiatrists). Diagnoses are not registered in The Danish Health Service Register for Primary Care; therefore, minor mental health conditions need to be identified in other ways. Talk therapy, psychometric tests given at GP, and contacts to psychologist are registered in The Danish Health Service Register for Primary Care and were used to identify minor mental health conditions. Furthermore, reimbursed prescriptions of antidepressant and anxiolytic medication were used to identify these conditions. Other psychotropic medication, for example antipsychotics, were not included due to broader indications such as nausea or analgesics.

Moderate to severe mental health conditions were identified as contact to a private psychiatrist or a registered psychiatric diagnosis (ICD-10 F00–99) at a psychiatric hospital. All mental health condition indicators had been measured within 5 years prior to the birth of the child. A reference group was defined as having no mental health-related contacts to GP, no contacts to psychologist or private psychiatrist, no reimbursed prescriptions of antidepressants or anxiolytic medication, and no registered psychiatric diagnosis within 5 years before the birth of the child (see Table 1).

**Table 1:** Criteria for exposure. Consensus definition of groups of mental conditions

Exposure	Specification *At least one criterion fulfilled*	Further criteria *All criteria fulfilled*	Healthcare sector and registry
*Minor mental health conditions*	**Medication** - At least 2 reimbursed prescriptions of: - antidepressant medicine (ATC N06AB, N06AX) - anxiolytic (benzodiazepines: ATC N03AE, N05BA, N05CD, N05CF) **Services at general practice** - At least two ‘talk therapy’ - At least two psychometric tests **Other services** - At least one contact to private psychologist	No contacts to psychiatric hospital and no psychiatric hospital diagnoses No records of contact to private psychiatrist	Primary healthcare sector The Danish Health Service Register for Primary Care Danish National Prescription Registry
*Moderate to severe mental health conditions*	- Any registered psychiatric diagnosis (ICD-10 F00–99) at psychiatric hospital - Mental health conditions treated at private psychiatrists (including child and adolescent psychiatrists)		Secondary healthcare sector The National Patient Register The Danish Health Service Register for Primary Care
Reference group *No mental health condition*	**None of above** - No registrations of psychiatric diagnoses and no mental health condition-related contacts to GP, psychologist or private psychiatrist **and** - No prescriptions of antidepressants or anxiolytic drugs		

Exposure was measured in a period of 5 years before the birth of the child

Two groups, minor and moderate-severe mental health conditions, and the unexposed reference group generated a matrix of exposures for the child due to the combinations of the mother and father in the different groups.

### Outcomes

All healthcare service contacts made within the first year of a child’s life were identified. Vaccinations and routine childcare visits were not included. Contact to GP daytime or out-of-hour service was defined as either consultation, telephone contact, or physical visit. Due to changes in the delivery of out-of-hours service in the Capital Region of Denmark in 2014, children from this region were excluded from the out-of-hour service analyses from 2014 to 2016. An inpatient contact was defined as any admission to hospital, and an outpatient hospital contact as any registered contact to an outpatient unit. Due to changes in registration of neonatal outpatient contacts during the study period, only outpatient contacts after the neonatal period (the first 28 days of life) were included. Any registered contact to the emergency department was defined as an ER contact.

### Covariates

Covariates, that could potentially confound the association between parental mental health conditions and child use of healthcare services were identified by reviewing the literature and a directed acyclic graph [29, 30] was constructed to hypothesize potential causal mechanisms (Supplementary figure 1). The same causal pathways were hypothesized for the different exposures and outcomes. Except for family income, which was extracted from the calendar year before the child’s birth, all covariates were extracted at the time of birth.

Based on the International Standard Classification of Education (ISCED 2011) [31], parental education was grouped according to highest completed education and divided into three groups: 1) Early childhood education (primary and lower secondary education (ISCED levels 0–2)); 2) General upper secondary education (high school programs, vocational upper secondary education, vocational training and education (ISCED 3–4)); and 3) Short-, medium-length or long-length higher education (first-, second- or third-cycle programs, tertiary education, bachelor or equivalent, Master’s or equivalent, Doctoral, PhD programs or the equivalent (5–8)).

Family income was defined as the equivalated disposable income for the family, which took into account the number of children and adults in the household. We adjusted for family income, parental age at time of birth of the child and calendar year using restricted cubic splines with three knots [32].

Family type was classified as either living with cohabiting parents or not.

Parity was divided into two groups according to whether the child was the mother’s first child or not.

Covariates related to birth outcomes, such as small for gestational age and birth weight, were considered mediators rather than confounders and were thus not adjusted for.

### Statistics

Baseline information was analyzed and reported as percentages based on maternal and paternal, respectively, mental health condition. In the following analyses, the maternal and paternal mental health condition groups were combined and a total of eight exposure groups and one reference group were generated. The reference group was both the mother and the father with no mental health condition. The eight exposed groups consisted of the different combinations of the mental health conditions of the father and/or the mother such as “father and mother minor mental health condition”, “father minor and mother moderate-severe mental health condition” and so on.

The number of each type of contacts, person-time in thousands of years and incidence rates were calculated for each exposure group and the reference group. Negative binomial regression was chosen due to overdispersion in the data. Both crude and adjusted negative binominal regression analyses were applied to the number of outcome events for each child during follow-up to estimate the incidence rate ratio (IRR) of experiencing the outcome. The adjusted regression analyses are complete case analyses and the covariates, that were adjusted for, are described in the Covariates section above.

Siblings appeared in the cohort and should have been treated in the analyses as dependent. A mixed effects model, however, was not possible to apply to such a large dataset. Instead, we made sensitivity analyses, which only included first-born children of mothers.

Non-fatal birth defects, prematurity and other significant conditions in early life might necessitate a higher number of healthcare contacts during the first year of life. To examine whether such conditions affected the estimates, a sensitivity analysis excluding children admitted for at least 1 week during first month of life was performed. Further, to examine whether the effects might differ over time a sensitivity analysis with different time periods was performed.

Data was analyzed using Stata SE 15.1.

## Results

**Fig. 1:**
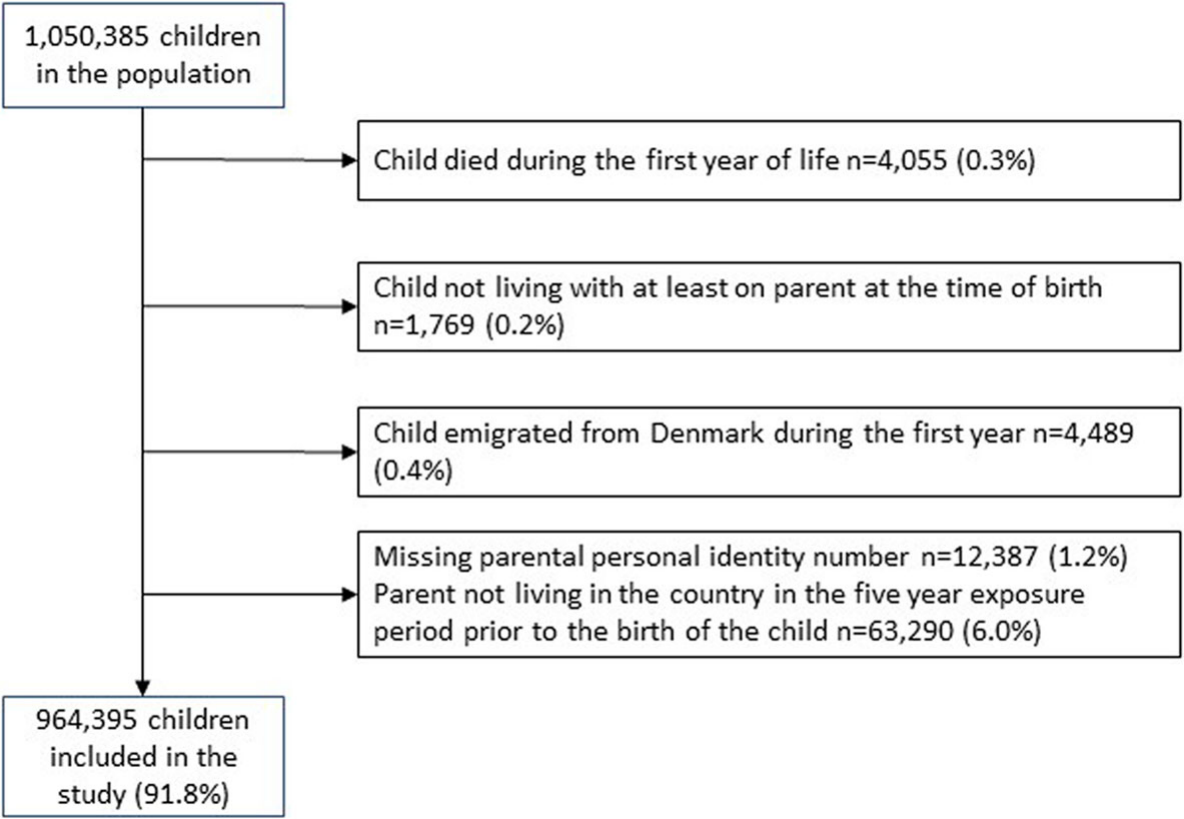
Flow chart of the study population

The cohort included 1,050,385 children. Children were excluded if they died during the first year of life, were not living with either of birth parents after birth or emigrated during the first year of life (0.9%). If parental personal identity number was missing or if a parent was not living in Denmark during the exposure period, the child was also excluded (7.2%). Characteristics of the excluded population is presented in the supplements section (Supplementary Table 1). In total, 964,395 children were included in the analyses (Fig. 1).

Educational level for both parents were lower for moderate-severe mental health condition when compared with both the reference group and minor mental health condition (Table 2). Parents with moderate-severe mental health conditions tended to be younger compared with both minor mental health conditions and the reference group. In 14% of mothers with minor mental health conditions, the corresponding father was also classified with minor mental health condition (22% in the case of the fathers). For mothers, 11 and 8% were classified with minor and moderate-severe, respectively, and for the fathers, the percentages were 8 and 5%, respectively.

**Table 2:** Baseline characteristics for maternal and paternal mental health condition (minor and moderate-severe) and reference group

	Mother			Father		
	Reference group (no mental health condition)	Minor	Moderate-severe	Reference group (no mental health condition)	Minor	Moderate-severe
**Children total N (%)**	773,734 (80.2)	111,040 (11.4)	79,621 (8.3)	843,353 (87.4)	72,551 (7.5)	48,491 (5.0)
Boys, percentages	51.3	51.4	51.4	51.3	51.3	51.9
Girls, percentages	48.7	48.6	48.6	48.7	48.7	48.1
**Parity, percentages**						
First child	42.9	43.4	48.3	43.3	42.4	47.2
Second or more	55.9	55.5	50.4	55.5	56.4	51.5
Missing	1.2	1.0	1.3	1.2	1.2	1.3
**Age, percentages**						
< 25	10.6	10.4	23.1	5.2	4.9	13.4
25–32	50.3	45.5	43.8	38.3	30.8	35.0
> 32	39.1	44.0	33.2	54.2	61.6	47.7
Missing	0.0	0.0	0.0	2.4	2.7	3.9
**Educational level, percentages**						
Low	14.6	17.3	35.8	16.4	22.9	41.0
Medium	38.6	38.0	36.0	47.1	43.5	37.6
High	45.9	44.0	27.3	35.3	32.1	18.3
Missing	0.9	0.6	1.0	1.1	1.5	3.1
**Household income, percentages**						
Low	32.6	27.8	37.8	31.9	31.5	43.2
Medium	37.4	36.4	28.1	37.5	33.7	23.3
High	19.9	22.0	11.9	20.1	19.6	9.0
Missing	10.1	13.8	22.2	10.4	15.1	24.4
**Living with cohabiting parents, percentages**						
Yes	94.5	91.3	83.9	94.2	90.0	81.0
No	5.0	8.2	15.4	5.2	9.5	18.3
Missing	0.6	0.5	0.7	0.6	0.5	0.6
**Mental health condition of the other parent, percentages**						
No mental health condition	90.1	78.8	74.2	82.6	65.0	61.5
Minor	6.1	14.4	11.8	10.4	22.1	15.5
Moderate-severe	3.9	6.8	14.0	7.0	12.9	23.0

The largest subgroup of the minor mental health condition group was characterized by contact to psychologist for the mothers (47.8%) and talk therapy at GP for the fathers (45.8%). In the moderate-severe mental health condition group, the most common diagnoses from psychiatric hospital was DF43–49 Other anxiety and stress-related disorders for the mothers (26.2%) and DF10–19 Mental and behavioral disorders due to psychoactive substance use for the fathers (31.6%). Number and percentages of mental healthcare service, prescriptions and diagnoses in the exposure groups are further described in the supplements (Supplementary Table 2).

The estimated incidence rates and incident rate ratios comparing the different exposure groups are presented in Tables 3 and 4. Overall, maternal mental health conditions yielded higher IRRs than paternal mental health conditions. For most outcomes, the IRRs were higher for moderate-severe than minor mental health conditions. The IRRs were smaller if only one parent had a mental health condition than if both parents had a mental health condition. The following three subsections summarize the results of these analyses. The crude IRRs are presented in Supplementary Table 3.

**Table 3:** Number of contacts, person-years per 1000 and incidence rates (95% confidence interval) for each exposure group

		Number of contacts			Person-years per 1000			Incidence rate (95% CI)		
**GP contacts daytime**										
		Mother								
Mental health condition		No mental health condition	Minor	Moderate-severe	No mental health condition	Minor	Moderate-severe	No mental health condition	Minor	Moderate-severe
Father	No mental health condition	670,155	85,229	57,631	697	87	59	961.76 (959.46–964.06)	974.34 (967.82–980.90)	975.54 (967.61–983.54)
Minor		45,543	15,647	9156	47	16	9	966.41 (957.57–975.33)	975.62 (960.45–991.03)	975.39 (955.62–995.58)
Moderate-severe		28,814	7353	10,877	30	8	11	966.75 (955.65–977.98)	976.75 (954.68–999.34)	974.82 (956.67–993.31)
**Out-of-hour contacts**										
No mental health condition		315,265	43,565	33,091	697	87	59	452.44 (450.87–454.03)	498.03 (493.38–502.73)	560.14 (554.14–566.21)
Minor		22,116	8092	5353	47	16	9	469.30 (463.15–475.52)	504.55 (493.68–515.67)	570.26 (555.18–585.74)
Moderate-severe		15,203	4055	6516	30	8	11	510.08 (502.04–518.26)	538.66 (522.33–555.49)	583.98 (569.97–598.33)
**ER contacts**										
No mental health condition		53,011	7831	6664	697	87	59	76.08 (75.43–76.73)	89.52 (87.56–91.53)	112.80 (110.13–115.54)
Minor		4111	1503	1071	47	16	9	87.23 (84.61–89.94)	93.71 (89.09–98.57)	114.09 (107.46–121.14)
Moderate-severe		3230	876	1406	30	8	11	108.37 (104.70–112.17)	116.37 (108.91–124.33)	126.01 (119.59–132.77)
**Inpatient contacts**										
No mental health condition		211,962	32,193	24,404	697	87	59	304.19 (302.90–305.49)	368.03 (364.03–372.07)	413.09 (407.94–418.31)
Minor		15,716	6194	4237	47	16	9	333.49 (328.32–338.74)	386.21 (376.71–395.95)	451.37 (437.98–465.17)
Moderate-severe		10,595	3021	5116	30	8	11	355.48 (348.77–362.31)	401.30 (387.24–415.87)	458.51 (446.11–471.24)
**Outpatient contacts**										
No mental health condition		151,273	24,191	17,425	697	87	59	217.10 (216.00–218.19)	276.55 (273.09–280.06)	294.96 (290.61–299.37)
Minor		11,927	4633	3146	47	16	9	253.09 (248.59–257.67)	288.88 (280.68–297.32)	335.14 (323.64–347.06)
Moderate-severe		7494	2243	3753	30	8	11	251.43 (245.81–257.19)	297.95 (285.88–310.54)	336.35 (325.76–347.29)

**Table 4:** Adjusted IRRs (95% CI) of each outcome for each group of mental health condition

**Adjusted IRRs (95% CI) of GP contacts daytime** for each combination of maternal and paternal mental health condition				
		Mother		
	Mental health condition	No mental health condition	Minor	Moderate-severe
Father	No mental health condition	Reference	1.17 (1.17–1.18)	1.18 (1.17–1.19)
	Minor	1.06 (1.05–1.07)	1.22 (1.21–1.24)	1.25 (1.23–1.27)
	Moderate-severe	1.05 (1.04–1.06)	1.17 (1.15–1.19)	1.18 (1.16–1.20)
**Adjusted IRRs (95% CI) of out-of-hour contacts** for each combination of maternal and paternal mental health condition *N* = 764,012 (84%)				
		Mother		
	Mental health condition	No mental health condition	Minor	Moderate-severe
Father	No mental health condition	Reference	1.26 (1.25–1.28)	1.39 (1.37–1.41)
	Minor	1.15 (1.14–1.17)	1.37 (1.34–1.41)	1.49 (1.45–1.54)
	Moderate-severe	1.20 (1.18–1.22)	1.36 (1.31–1.41)	1.44 (1.40–1.49)
**Adjusted IRRs (95% CI) of ER contacts** for each combination of maternal and paternal mental health condition *N* = 810,478 (84%)				
		Mother		
	Mental health condition	No mental health condition	Minor	Moderate-severe
Father	No mental health condition	Reference	1.14 (1.11–1.18)	1.36 (1.31–1.41)
	Minor	1.14 (1.10–1.19)	1.25 (1.17–1.34)	1.39 (1.27–1.52)
	Moderate-severe	1.31 (1.25–1.38)	1.27 (1.15–1.41)	1.35 (1.24–1.47)
**Adjusted IRRs (95% CI) of inpatient contacts** for each combination of maternal and paternal mental health condition *N* = 810,478 (84%)				
		Mother		
	Mental health condition	No mental health condition	Minor	Moderate-severe
Father	No mental health condition	Reference	1.25 (1.24–1.27)	1.39 (1.36–1.41)
	Minor	1.11 (1.08–1.13)	1.33 (1.29–1.37)	1.48 (1.43–1.54)
	Moderate-severe	1.12 (1.10–1.15)	1.26 (1.21–1.32)	1.41 (1.36–1.47)
**Adjusted IRRs (95% CI) of outpatient contact** for each combination of maternal and paternal mental health condition *N* = 810,478 (84%)				
		Mother		
	Mental health condition	No mental health condition	Minor	Moderate-severe
Father	No mental health condition	Reference	1.22 (1.19–1.25)	1.29 (1.25–1.33)
	Minor	1.07 (1.04–1.11)	1.27 (1.20–1.35)	1.59 (1.47–1.72)
	Moderate-severe	1.12 (1.07–1.17)	1.21 (1.10–1.32)	1.47 (1.36–1.59)

Adjusted for calendar year, sex, parental age, parental education, family income, family type and parity

Complete case analysis based on 84% of the study population

*IRR* Incidence rate ratio

*CI* Confidence interval

### Mother with mental health conditions

Children of mothers with mental health conditions (and fathers with no mental health condition) had higher incidence rates of all types of healthcare contacts than children of both parents with no mental health condition (Table 3). Further, the incidence rates for moderate-severe mental health conditions were higher than for minor mental health conditions for all outcomes; e.g. for no, minor and moderate-severe mental health conditions the incidence rates for out-of-hour contacts were 452 (95% CI 451–454), 498 (95% CI 493–503) and 560 (95% CI 554–566) contacts per 1000 person years.

Adjusted negative binominal regression analyses (Table 4) revealed higher incidence compared to the reference group for all outcomes ranging from 14% (IRR 1.14 (95% CI 1.11–1.18)) for ER contacts among children with maternal minor mental health conditions to 39% (IRR 1.39 (95% CI 1.37–1.41)) for out-of-hour contacts in children with maternal moderate-severe mental health conditions. The IRRs of maternal moderate-severe mental health conditions were higher than minor mental health conditions for all outcomes.

### Father with mental health conditions

Children of fathers with mental health conditions (and mothers with no mental health conditions) also had higher incidence rates of all outcomes than children with both parents with no mental health condition. However, this result was less evident than for the maternal mental health conditions (Table 3). For all outcomes except outpatient contacts the incidence rates were higher for moderate-severe mental health conditions than for minor mental health conditions; e.g. for no, minor and moderate-severe mental health conditions the incidence rates for out-of-hour contacts were 452 (95% CI 451–454), 469 (95% CI 463–476) and 510 (95% CI 502–518) contacts per 1000 person years.

The IRRs of the adjusted analyses also showed increased incidence rates for all outcomes, but less than for maternal exposure (Table 4). The relative increase in incidence ranged from 5% (IRR 1.05 (95% CI 1.04–1.06)) for GP contacts to 31% (IRR 1.31 (95% CI 1.25–1.38)) for ER contacts for children of fathers with moderate-severe mental health conditions. For all outcomes but GP contacts, the IRRs were higher for moderate-severe than for minor mental health conditions.

### Both parents with mental health conditions

For all outcomes but one, the incidence rates were higher for children of parents who both had mental health conditions than for children with only one parent with mental health conditions (Table 3). The only exception was for maternal moderate-severe mental health condition and GP contacts, where the incidence rates were the same regardless of the mental health condition of the father.

The adjusted analyses found the IRRs higher for children with both parents with mental health conditions than only one parent with mental health condition (Table 4). The exceptions were one estimate of outpatient contacts (mother with minor and father with moderate-severe mental health condition) and two estimates of ER contacts (father with moderate-severe mental health condition).

Compared to the reference group, the relative increase in incidence for outpatient contacts ranged from 21% (IRR 1.21 (95% 1.10–1.32)) for children of fathers with moderate-severe and mothers with minor mental health conditions to 59% (IRR 1.59 (95% CI 1.47–1.72)) for children of father with minor and mothers with moderate-severe mental health conditions. When both parents had moderate-severe mental health conditions, the incidence of outpatient contacts was higher by 47% (IRR 1.47 (95% CI 1.36–1.59)).

The sensitivity analyses including only first-born children (*n* = 418,785) did not change the estimates markedly (Supplementary Table 4). Neither did the sensitivity analyses (Supplementary Table 5) excluding children with at least 1 week of admission during the first month of life (940,397 children included in the analyses). The sensitivity analyses in different time periods showed no trend nor systematic change of the estimates over time (Supplementary Table 6).

## Discussion

Both maternal and paternal mental health conditions were independently associated with child use of healthcare services during the first year of life. These associations were also found for minor mental health conditions only handled in primary care with the risk being higher if both parents had a mental health condition.

### Interpretation

A range of factors might explain the association between parental mental health conditions and increased use of healthcare services in infants. These factors are related to parental healthcare behavior, healthcare service-related factors and the health of the child. First, it has been argued that mothers with depression/anxiety are more likely to seek medical care for their child’s minor injuries [11]. Furthermore, prenatal anxiety was associated with less parenting self-efficacy [33] and higher levels of stress [33, 34] which could consequently lead to increasing parental need of medical guidance and assessment.

Second, health professionals might be more likely to schedule extra contacts for the children of vulnerable parents [35]. In these cases, increased use of healthcare services might not necessarily be negative. Third, children of parents with poor mental health might be more likely to have poor health. This has been confirmed by a recent systematic review and meta-analysis [16]. A Danish study found a higher use of primary healthcare as well as a higher rate of positive tests (C-reactive protein, Strep A test, Urinary stix) in children of mothers with depression which could be interpreted as a higher rate of infectious disease [2].

Mental health conditions of the father appeared to also influence the utilization of healthcare services. This was particularly found to be the case if the mother was also classified as having mental health conditions. Conversely, having one parent without a mental health condition reduced the impact of a mental health condition in the other parent which illustrates a possible protective impact of the ‘mentally healthy’ parent.

The risks of GP and inpatient contacts did not differentiate whether the father was classified with minor or moderate-severe mental health condition and only minor differences were seen for outpatient contacts. This was also the case for maternal health conditions and GP contacts. These findings could indicate that mental vulnerability in general and not severity of the mental health condition influences the use of healthcare services.

### Implications for policy, practice and research

This study showed an association between parental mental health condition and the use of healthcare services by a child younger than 12 months. This highlights the important role of the father’s mental health which is often overlooked in research [16] and in society generally [36].

In Denmark, minor mental health conditions are only handled in primary care and might get less public and political attention compared to more severe mental illness. This study revealed an association between minor mental health conditions in parents and increased use of healthcare in infants for all categories of healthcare services. It is noteworthy that the estimates for some of the outcomes did not differ based on whether either parent was classified with a minor or moderate/major mental health condition. These findings point to a greater need for focus on minor mental health conditions of both mothers as and fathers in the planning of services in pre- and postnatal care and throughout the first year of a child’s life.

Although the effects were modest, we find our results of public health relevance. As approximately on fifth of this nationally representative sample was exposed to maternal mental health condition, even a modest increased effect might produce a high burden at population level and be of public health relevance.

Further research is needed to explore the mechanisms behind parental mental health conditions and the use of healthcare services and should examine whether children of parents with mental health conditions have a greater incidence of morbidities.

### Strengths and limitations

This study has a couple of strengths: 1) The nationwide registries on an entire birth cohort in Denmark allows for powerful statistical analyses, and 2) the Danish registries are considered valid in terms of completeness, registration processes and accuracy [21–23, 37].

This study also has limitations. There is a risk of misclassification of minor parental mental health conditions as we could not access diagnoses or indication of prescribed medication, talk therapy or the results of the psychometric tests from The Danish Health Service Register for Primary Care. Furthermore, we only identified parents with poor mental health who sought medical care. Thus, parents with poor mental health who had no mental health condition-related contacts to GP, private psychologist, prescribed antidepressants or anxiolytic drugs or contacts to psychiatrist or psychiatric hospital were misclassified as being in the reference group. Moreover, parents with a psychiatric disorder who had no records of psychiatric hospital admission in the 5 years prior to birth of the child were also misclassified as being in the reference group. The above-mentioned possible misclassifications may cause an underestimation of the association between parental mental health conditions and infant healthcare use.

The cohort included births over several years and hence included many calendar years in the follow-up period. This led to many structural changes during the follow-up period e.g. changes of guidelines and procedures of registration. For example in 2009, the guidelines for the perinatal care in Denmark were updated with recommendations of differentiated care [38]. That implied new recommendations of level division of the perinatal care and cross-sectional cooperation in order to ensure the right support and care for the pregnant women regarding obstetric as well as social and mental risk factors. Both exposure groups, especially minor mental health conditions, were markedly smaller in the first years of the study period. These numbers increased throughout the period of this study (data not shown). This increase was also noted by the Danish Health Authority that described a three-percentage point increase of the prevalence of poor mental health in Danish adults from 2010 to 2017 [39]. This issue was handled in the analyses by controlling for calendar year and further tested in a sensitivity analysis with different time periods (Supplementary Table 6).

## Conclusion

Both maternal and paternal minor as well as moderate/severe mental health conditions were associated with an increased use of all types of healthcare services for the child during the first year of life. The risk was greater if both parents had a mental health condition. These findings point to a greater focus on both parents’ minor mental health conditions in the planning and delivery of healthcare services.

## Supplementary Information

**Additional file 1: Supplementary Table 1.** Characteristics of the excluded population.

**Additional file 2: Supplementary Table 2.** Number and percentages in each exposure group and subgroup.

**Additional file 3: Supplementary Table 3.** Crude incidence rate ratio (95% confidence interval) of healthcare contacts for each exposure group.

**Additional file 4: Supplementary Table 4.** Sensitivity analysis only including first-born. Incidence rate ratio (95% confidence interval), crude and adjusted, of healthcare contacts for each exposure group.

**Additional file 5: Supplementary Table 5.** Sensitivity analysis excluding children with at least one week of hospital admission in the first month of life. Incidence rate ratio (95% confidence interval), crude and adjusted, of healthcare contacts for each exposure group.

**Additional file 6: Supplementary Table 6.** Adjusted incidence rate ratios (95% confidence interval) of each outcome in strata of calendar year.

**Additional file 7: Supplementary figure 1.** Directed acyclic graph.

## Data Availability

The data that support the findings of this study is available from the Statistics Denmark but restrictions apply to the availability of these data, which were used under license for the current study, and so are not publicly available. Questions or requests concerning this data is directed to the corresponding author Signe Heuckendorff.

## Abbreviations

CI: Confidence interval
ER: Emergency room
GP: General practitioner
IRR: Incidence rate ratio
