# Supplementary material for: Parental mental health conditions and use of healthcare services in children the first year of life– a register-based, nationwide study
Source: BMC Public Health. 2021 Mar 21;21:557. doi: 10.1186/s12889-021-10625-y (PMC7981963; doi:10.1186/s12889-021-10625-y)
Supplement: Supplementary file 1 — Additional file 1: Supplementary Table 1. Characteristics of the excluded population. [file 12889_2021_10625_MOESM1_ESM.docx]

### **Supplementary table 1. Characteristics of the excluded population**

| \|  \| **Mother** \| \| \|  \| **Father** \| \| \| \| --- \| --- \| --- \| --- \| --- \| --- \| --- \| --- \| \|  \| **No mental health condition** \| **Minor** \| **Moderate-severe** \|  \| **No mental health condition** \| **Minor** \| **Moderate-severe** \| \| **Children total N (%)** \| 75044 (87.3) \| 5404 (6.3) \| 5542 (6.4) \|  \| 65544 (90.1) \| 3809 (5.2) \| 3412 (4.7) \| \| Boys, percentages \| 51.3 \| 52.1 \| 51.8 \|  \| 51.5 \| 51.5 \| 52.1 \| \| Girls, percentages \| 48.6 \| 47.9 \| 48.1 \|  \| 48.5 \| 48.4 \| 47.9 \| \| **Parity** \| \| \| \| \| \| \| \| \| First child \| 58.0 \| 55.9 \| 55.8 \|  \| 56.8 \| 53.7 \| 52.3 \| \| Second or more \| 40.3 \| 42.8 \| 42.2 \|  \| 41.5 \| 44.7 \| 46.3 \| \| Missing \| 1.7 \| 1.4 \| 2.0 \|  \| 1.6 \| 1.7 \| 1.4 \| \| **Age** \| \| \| \| \| \| \| \| \| <25 \| 20.5 \| 11.5 \| 25.9 \|  \| 6.7 \| 3.0 \| 7.6 \| \| 25-32 \| 46.0 \| 36.1 \| 37.7 \|  \| 30.6 \| 20.8 \| 25.1 \| \| >32 \| 33.1 \| 52.4 \| 36.2 \|  \| 43.1 \| 61.5 \| 56.7 \| \| Missing \| 0.4 \| 0.0 \| 0.1 \|  \| 19.6 \| 14.6 \| 10.7 \| \| **Educational level** \| \| \| \| \| \| \| \| \| Low \| 33.5 \| 26.2 \| 47.4 \|  \| 27.9 \| 30.3 \| 43.4 \| \| Medium \| 26.2 \| 32.1 \| 27.3 \|  \| 28.1 \| 33.9 \| 30.1 \| \| High \| 28.1 \| 37.5 \| 19.8 \|  \| 27.0 \| 27.4 \| 18.4 \| \| Missing \| 12.2 \| 4.2 \| 5.5 \|  \| 17.1 \| 8.5 \| 8.1 \| \| **Household income** \| \| \| \| \| \| \| \| \| Low \| 48.5 \| 39.9 \| 49.4 \|  \| 48.4 \| 48.5 \| 56.9 \| \| Medium \| 12.3 \| 17.7 \| 10.1 \|  \| 11.8 \| 11.8 \| 6.2 \| \| High \| 6.5 \| 9.3 \| 3.3 \|  \| 6.5 \| 6.1 \| 2.1 \| \| Missing \| 32.7 \| 33.1 \| 37.2 \|  \| 33.3 \| 33.6 \| 34.8 \| \| **Living with cohabiting parents** \| \| \| \| \| \| \| \| \| Yes \| 62.0 \| 41.6 \| 36.4 \|  \| 69.6 \| 72.7 \| 70.2 \| \| No \| 2.2 \| 3.5 \| 4.7 \|  \| 2.7 \| 4.1 \| 5.2 \| \| Missing \| 35.8 \| 54.8 \| 58.9 \|  \| 27.7 \| 23.2 \| 24.6 \| \| **Mental health condition of the other parent** \| \| \| \| \| \| \| \| \| No mental health condition \| 79.3 \| 56.8 \| 53.3 \|  \| 90.8 \| 83.0 \| 82.7 \| \| Minor \| 4.2 \| 6.4 \| 5.5 \|  \| 4.7 \| 9.1 \| 5.8 \| \| Moderate-severe \| 3.8 \| 3.6 \| 7.1 \|  \| 4.5 \| 8.0 \| 11.5 \| |  |  |  |  |
| --- | --- | --- | --- | --- | --- | --- | --- | --- | --- | --- | --- | --- | --- | --- | --- | --- | --- | --- | --- | --- | --- | --- | --- | --- | --- | --- | --- | --- | --- | --- | --- | --- | --- | --- | --- | --- | --- | --- | --- | --- | --- | --- | --- | --- | --- | --- | --- | --- | --- | --- | --- | --- | --- | --- | --- | --- | --- | --- | --- | --- | --- | --- | --- | --- | --- | --- | --- | --- | --- | --- | --- | --- | --- | --- | --- | --- | --- | --- | --- | --- | --- | --- | --- | --- | --- | --- | --- | --- | --- | --- | --- | --- | --- | --- | --- | --- | --- | --- | --- | --- | --- | --- | --- | --- | --- | --- | --- | --- | --- | --- | --- | --- | --- | --- | --- | --- | --- | --- | --- | --- | --- | --- | --- | --- | --- | --- | --- | --- | --- | --- | --- | --- | --- | --- | --- | --- | --- | --- | --- | --- | --- | --- | --- | --- | --- | --- | --- | --- | --- | --- | --- | --- | --- | --- | --- | --- | --- | --- | --- | --- | --- | --- | --- | --- | --- | --- | --- | --- | --- | --- | --- | --- | --- | --- | --- | --- | --- | --- | --- | --- | --- | --- | --- | --- | --- | --- | --- | --- | --- | --- | --- | --- | --- | --- | --- | --- | --- | --- | --- | --- | --- | --- | --- | --- | --- | --- | --- | --- | --- | --- | --- | --- | --- | --- | --- | --- | --- | --- | --- | --- | --- | --- | --- | --- | --- | --- | --- | --- | --- | --- | --- | --- | --- | --- | --- | --- | --- | --- | --- | --- | --- | --- | --- | --- | --- | --- | --- | --- | --- | --- | --- | --- | --- | --- | --- | --- | --- | --- | --- | --- |

### 
