# Supplementary material for: Parental mental health conditions and use of healthcare services in children the first year of life– a register-based, nationwide study
Source: BMC Public Health. 2021 Mar 21;21:557. doi: 10.1186/s12889-021-10625-y (PMC7981963; doi:10.1186/s12889-021-10625-y)
Supplement: Supplementary file 2 — Additional file 2: Supplementary Table 2. Number and percentages in each exposure group and subgroup. [file 12889_2021_10625_MOESM2_ESM.docx]

### **Supplementary table 2. Number and percentages in each exposure group and subgroup**

|  | | |
| --- | --- | --- |
| NB: The same parent might appear in multiple subgroups | |  |
|  |  |  |
| **Minor mental health conditions** |  |  |
| Number and percentages in the subgroup out of the group with minor mental health conditions | |  |
|  | Mother | Father |
| Prescriptions of SSRI or other antidepressants (N06AB, N06AX) | 33213 (29.91) | 23145 (31.90) |
| Prescriptions of benzodiazepines (ATC N03AE, N05BA, N05CD, N05CF) | 17074 (15.38) | 17151 (23.64) |
| Psychometric test at GP | 18331 (16.51) | 9321 (12.85) |
| Supportive conversations/therapy talk at GP | 38584 (34.75) | 33198 (45.76) |
| Contact to private psychologist | 53025 (47.75) | 19862 (27.38) |
|  |  |  |
| **Moderate-severe mental health conditions** |  |  |
| Number and percentages in the subgroup out of the group with moderate-severe mental health conditions | |  |
|  | Mother | Father |
| At least one contact to private psychiatrist | 25278 (31.75) | 17034 (35.13) |
| F10-19, Mental and behavioural disorders due to psychoactive substance use | 14144 (17.76) | 15339 (31.63) |
| F20-29, Schizophrenia spectrum disorder | 2818 (3.54) | 3066 (6.32) |
| F30-31, Bipolar disorder | 1182 (1.48) | 726 (1.50) |
| F32-34, Unipolar depression | 18559 (23.31) | 6596 (13.60) |
| F35-39, Other affective disorders | 451 (0.57) | 162 (0.33) |
| F40-41, Anxiety disorders | 11251 (14.13) | 3696 (7.62) |
| F42, Obsessive compulsive disorder | 2002 (2.51) | 673 (1.39) |
| F43-49, Other anxiety and stress-related disorders | 20819 (26.15) | 10907 (22.49) |
| F50, Eating disorders | 4923 (6.18) | 97 (0.20) |
| F60-62, Personality disorders | 11038 (13.86) | 4356 (8.98) |
| F70-79, Intellectual disabilities | 418 (0.52) | 250 (0.52) |
| Other F-diagnoses, F00-09,51-59,63-69,80-99 | 8275 (10.39) | 7015 (14.47) |
