# Supplementary material for: Parental mental health conditions and use of healthcare services in children the first year of life– a register-based, nationwide study
Source: BMC Public Health. 2021 Mar 21;21:557. doi: 10.1186/s12889-021-10625-y (PMC7981963; doi:10.1186/s12889-021-10625-y)
Supplement: Supplementary file 3 — Additional file 3: Supplementary Table 3. Crude incidence rate ratio (95% confidence interval) of healthcare contacts for each exposure group. [file 12889_2021_10625_MOESM3_ESM.docx]

### **Supplementary table 3. Crude incidence rate ratio (95% confidence interval) of healthcare contacts for each exposure group**

| **GP contacts daytime, crude N = 964395** | | |  |  |
| --- | --- | --- | --- | --- |
|  |  | Mother | IRR (CI95) |  |
|  | Mental health condition | No mental health condition | Minor | Moderate-severe |
| Father | No mental health condition | Reference | 1.16 (1.16 - 1.17) | 1.19 (1.18 - 1.20) |
|  | Minor | 1.05 (1.04 - 1.05) | 1.19 (1.18 - 1.20) | 1.23 (1.22 - 1.25) |
|  | Moderate-severe | 1.05 (1.04 - 1.06) | 1.17 (1.15 - 1.19) | 1.19 (1.18 - 1.21) |
|  |  |  |  |  |
| **Out-of hour contacts, crude N = 909358** | | |  |  |
|  |  | Mother |  |  |
|  | Mental health condition | No mental health condition | Minor | Moderate-severe |
| Father | No mental health condition | Reference | 1.22 (1.22 - 1.24) | 1.55 (1.53 - 1.56) |
|  | Minor | 1.12 (1.10 - 1.13) | 1.28 (1.26 - 1.31) | 1.62 (1.58 - 1.66) |
|  | Moderate-severe | 1.34 (1.32 - 1.36) | 1.50 (1.45 - 1.54) | 1.72 (1.68 - 1.77) |
|  |  |  |  |  |
| **ER contacts, crude N = 964395** | | | | |
|  |  | Mother |  |  |
|  | Mental health condition | No metal health condition | Minor | Moderate-severe |
| Father | No mental health condition | Reference | 1.21 (1.17 - 1.24) | 1.62 (1.58 - 1.68) |
|  | Minor | 1.17 (1.12 - 1.21) | 1.28 (1.20 - 1.36) | 1.69 (1.57 - 1.82) |
|  | Moderate-severe | 1.52 (1.46 - 1.59) | 1.60 (1.47 - 1.74) | 1.88 (1.76 - 2.01) |
|  |  |  |  |  |
| **Inpatient contacts, crude N = 964395** | | |  |  |
|  |  | Mother |  |  |
|  | Mental health condition | No mental health condition | Minor | Moderate-severe |
| Father | No mental health condition | Reference | 1.30 (1.28 - 1.32) | 1.53 (1.51 - 1.55) |
|  | Minor | 1.14 (1.12 - 1.16) | 1.38 (1.35 - 1.42) | 1.68 (1.63 - 1.74) |
|  | Moderate-severe | 1.26 (1.23 - 1.28) | 1.47 (1.41 - 1.52) | 1.72 (1.67 - 1.77) |
|  |  |  |  |  |
| **Outpatient contacts, crude N = 964395** | | |  |  |
|  |  | Mother |  |  |
|  | Mental health condition | No mental health condition | Minor | Moderate-severe |
| Father | No mental health condition | Reference | 1.29 (1.26 - 1.32) | 1.46 (1.42 - 1.50) |
|  | Minor | 1.15 (1.11 - 1.19) | 1.41 (1.33 - 1.48) | 1.89 (1.77 - 2.02) |
|  | Moderate-severe | 1.24 (1.19 - 1.29) | 1.43 (1.33 - 1.54) | 1.89 (1.78 - 2.01) |
