# Supplementary material for: Parental mental health conditions and use of healthcare services in children the first year of life– a register-based, nationwide study
Source: BMC Public Health. 2021 Mar 21;21:557. doi: 10.1186/s12889-021-10625-y (PMC7981963; doi:10.1186/s12889-021-10625-y)
Supplement: Supplementary file 4 — Additional file 4: Supplementary Table 4. Sensitivity analysis only including first-born. Incidence rate ratio (95% confidence interval), crude and adjusted, of healthcare contacts for each exposure group. [file 12889_2021_10625_MOESM4_ESM.docx]

### **Supplementary table 4. Sensitivity analyses only including first-born. Incidence rate ratio (95% confidence interval), crude and adjusted, of healthcare contacts for each exposure group**

| Unadjusted | |  |  |  |
| --- | --- | --- | --- | --- |
|  |  |  |  |  |
| Number of children | | 418745 |  |  |
|  |  |  | IRR (CI95) |  |
| **GP contacts daytime, crude** | | | | |
|  |  | Mother | | |
|  | Mental health condition | No mental health condition | Minor | Moderate-severe |
| Father | No mental health condition | Reference | 1.16 (1.15 - 1.17) | 1.17 (1.17 - 1.18) |
|  | Minor | 1.05 (1.04 - 1.06) | 1.18 (1.16 - 1.20) | 1.23 (1.20 - 1.25) |
|  | Moderate-severe | 1.04 (1.03 - 1.05) | 1.18 (1.15 - 1.21) | 1.19 (1.17 - 1.21) |
|  |  |  |  |  |
| **Out-of hour contacts, crude** | | | | |
|  |  | Mother | | |
|  | Mental health condition | No mental health condition | Minor | Moderate-severe |
| Father | No mental health condition | Reference | 1.21 (1.19 - 1.23) | 1.55 (1.52 - 1.57) |
|  | Minor | 1.10 (1.08 - 1.13) | 1.24 (1.19 - 1.28) | 1.63 (1.56 - 1.69) |
|  | Moderate-severe | 1.35 (1.32 - 1.38) | 1.55 (1.48 - 1.62) | 1.78 (1.72 - 1.84) |
|  |  |  |  |  |
| **ER contacts, crude** | | | | |
|  |  | Mother | | |
|  | Mental health condition | No mental health condition | Minor | Moderate-severe |
| Father | No mental health condition | Reference | 1.19 (1.15 - 1.24) | 1.64 (1.57 - 1.71) |
|  | Minor | 1.15 (1.09 - 1.21) | 1.34 (1.22 - 1.47) | 1.72 (1.55 - 1.91) |
|  | Moderate-severe | 1.48 (1.39 - 1.57) | 1.58 (1.40 - 1.78) | 1.91 (1.75 - 2.10) |
|  |  |  |  |  |
| **Inpatient contacts, crude** | | | | |
|  |  | Mother | | |
|  | Mental health condition | No mental health condition | Minor | Moderate-severe |
| Father | No mental health condition | Reference | 1.27 (1.25 - 1.29) | 1.46 (1.44 - 1.49) |
|  | Minor | 1.14 (1.11 - 1.17) | 1.35 (1.29 - 1.40) | 1.61 (1.53 - 1.68) |
|  | Moderate-severe | 1.21 (1.17 - 1.24) | 1.46 (1.39 - 1.54) | 1.63 (1.56 - 1.70) |
|  |  |  |  |  |
| **Outpatient contacts, crude** | | | | |
|  |  | Mother | | |
|  | Mental health condition | No mental health condition | Minor | Moderate-severe |
| Father | No mental health condition | Reference | 1.29 (1.25 - 1.33) | 1.42 (1.37 - 1.48) |
|  | Minor | 1.15 (1.10 - 1.21) | 1.42 (1.31 - 1.54) | 1.87 (1.70 - 2.05) |
|  | Moderate-severe | 1.13 (1.07 - 1.19) | 1.48 (1.33 - 1.65) | 1.77 (1.63 - 1.93) |

| Adjusted for calendar year, sex, parental age, parental education, family income and family type | | | | |
| --- | --- | --- | --- | --- |
|  |  |  |  |  |
| **GP contacts daytime, adjusted** | | | | |
|  |  | Mother | | |
|  | Mental health condition | No mental health condition | Minor | Moderate-severe |
| Father | No mental health condition | Reference | 1.16 (1.16 - 1.17) | 1.17 (1.16 - 1.18) |
|  | Minor | 1.07 (1.06 - 1.08) | 1.19 (1.17 - 1.22) | 1.24 (1.21 - 1.27) |
|  | Moderate-severe | 1.04 (1.03 - 1.06) | 1.16 (1.13 - 1.20) | 1.17 (1.14 - 1.20) |
|  |  |  |  |  |
| **Out-of hour contacts, adjusted** | | | | |
|  |  | Mother | | |
|  | Mental health condition | No mental health condition | Minor | Moderate-severe |
| Father | No mental health condition | Reference | 1.25 (1.23 - 1.27) | 1.35 (1.33 - 1.38) |
|  | Minor | 1.15 (1.12 - 1.17) | 1.33 (1.28 - 1.38) | 1.48 (1.41 - 1.55) |
|  | Moderate-severe | 1.19 (1.16 - 1.22) | 1.39 (1.32 - 1.47) | 1.40 (1.33 - 1.46) |
|  |  |  |  |  |
| **ER contacts, adjusted** | | | | |
|  |  | Mother | | |
|  | Mental health condition | No mental health condition | Minor | Moderate-severe |
| Father | No mental health condition | Reference | 1.13 (1.08 - 1.18) | 1.37 (1.30 - 1.45) |
|  | Minor | 1.14 (1.07 - 1.21) | 1.28 (1.16 - 1.43) | 1.37 (1.20 - 1.57) |
|  | Moderate-severe | 1.28 (1.19 - 1.38) | 1.25 (1.07 - 1.45) | 1.33 (1.17 - 1.51) |
|  |  |  |  |  |
| **Inpatient contacts, adjusted** | | | | |
|  |  | Mother | | |
|  | Mental health condition | No mental health condition | Minor | Moderate-severe |
| Father | No mental health condition | Reference | 1.23 (1.21 - 1.26) | 1.34 (1.30 - 1.37) |
|  | Minor | 1.11 (1.08 - 1.14) | 1.28 (1.22 - 1.34) | 1.41 (1.33 - 1.49) |
|  | Moderate-severe | 1.11 (1.07 - 1.15) | 1.30 (1.22 - 1.39) | 1.38 (1.30 - 1.46) |
|  |  |  |  |  |
| **Outpatient contacts, adjusted** | | | | |
|  |  | Mother | | |
|  | Mental health condition | No mental health condition | Minor | Moderate-severe |
| Father | No mental health condtion | Reference | 1.22 (1.17 - 1.26) | 1.29 (1.23 - 1.35) |
|  | Minor | 1.09 (1.03 - 1.14) | 1.26 (1.15 - 1.37) | 1.45 (1.29 - 1.63) |
|  | Moderate-severe | 1.06 (0.99 - 1.13) | 1.23 (1.08 - 1.41) | 1.39 (1.24 - 1.55) |
