# Supplementary material for: Parental mental health conditions and use of healthcare services in children the first year of life– a register-based, nationwide study
Source: BMC Public Health. 2021 Mar 21;21:557. doi: 10.1186/s12889-021-10625-y (PMC7981963; doi:10.1186/s12889-021-10625-y)
Supplement: Supplementary file 5 — Additional file 5: Supplementary Table 5. Sensitivity analysis excluding children with at least one week of hospital admission in the first month of life. Incidence rate ratio (95% confidence interval), crude and adjusted, of healthcare contacts for each exposure group. [file 12889_2021_10625_MOESM5_ESM.docx]

### **Supplementary table 5. Sensitivity analyses excluding children with at least one week of hospital admission in the first month of life. Incidence rate ratio (95% confidence interval), crude and adjusted, of healthcare contacts for each exposure group**

| Unadjusted | |  |  |  |
| --- | --- | --- | --- | --- |
|  |  |  |  |  |
| Number of children | | 940397 |  |  |
|  |  |  | IRR (CI95) |  |
| **GP contacts daytime, crude** | | |  |  |
|  |  | Mother |  |  |
|  | Mental health condition | No mental health condition | Minor | Moderate-severe |
| Father | No mental health condition | Reference | 1.16 (1.16 - 1.17) | 1.19 (1.18 - 1.20) |
|  | Minor | 1.05 (1.04 - 1.05) | 1.19 (1.18 - 1.21) | 1.23 (1.22 - 1.25) |
|  | Moderate-severe | 1.05 (1.04 - 1.06) | 1.17 (1.15 - 1.19) | 1.20 (1.18 - 1.21) |
|  |  |  |  |  |
| **Out-of hour contacts, crude** | | |  |  |
|  |  | Mother |  |  |
|  | Mental health condition | No mental health condition | Minor | Moderate-severe |
| Father | No mental health condition | Reference | 1.23 (1.21 - 1.24) | 1.55 (1.53 - 1.56) |
|  | Minor | 1.12 (1.10 - 1.13) | 1.29 (1.26 - 1.32) | 1.62 (1.58 - 1.67) |
|  | Moderate-severe | 1.34 (1.31 - 1.36) | 1.50 (1.45 - 1.55) | 1.73 (1.69 - 1.77) |
|  |  |  |  |  |
| **ER contacts, crude** | |  |  |  |
|  |  | Mother |  |  |
|  | Mental health condition | No mental health condition | Minor | Moderate-severe |
| Father | No mental health condition | Reference | 1.21 (1.18 - 1.25) | 1.63 (1.58 - 1.68) |
|  | Minor | 1.17 (1.13 - 1.22) | 1.27 (1.20 - 1.35) | 1.70 (1.58 - 1.83) |
|  | Moderate-severe | 1.53 (1.46 - 1.60) | 1.60 (1.47 - 1.74) | 1.89 (1.77 - 2.03) |
|  |  |  |  |  |
| **Inpatient contacts, crude** | |  |  |  |
|  |  | Mother |  |  |
|  | Mental health condition | No mental health condition | Minor | Moderate-severe |
| Father | No mental health condition | Reference | 1.32 (1.30 - 1.34) | 1.54 (1.52 - 1.57) |
|  | Minor | 1.15 (1.13 - 1.17) | 1.39 (1.35 - 1.43) | 1.68 (1.62 - 1.74) |
|  | Moderate-severe | 1.26 (1.24 - 1.29) | 1.49 (1.44 - 1.55) | 1.72 (1.67 - 1.77) |
|  |  |  |  |  |
| **Outpatient contacts, crude** | | |  |  |
|  |  | Mother |  |  |
|  | Mental health condition | No mental health condition | Minor | Moderate-severe |
| Father | No mental health condition | Reference | 1.31 (1.28 - 1.35) | 1.46 (1.42 - 1.51) |
|  | Minor | 1.16 (1.13 - 1.20) | 1.39 (1.32 - 1.47) | 1.83 (1.71 - 1.96) |
|  | Moderate-severe | 1.22 (1.18 - 1.27) | 1.47 (1.36 - 1.58) | 1.86 (1.75 - 1.98) |

| Adjusted for calendar year, sex, parental age, parental education and family type | | | | |
| --- | --- | --- | --- | --- |
| **GP contacts daytime, adjusted** | | |  |  |
|  |  | Mother |  |  |
|  | Mental health condition | No mental health condition | Minor | Moderate-severe |
| Father | No mental health condition | Reference | 1.18 (1.17 - 1.18) | 1.18 (1.17 - 1.19) |
|  | Minor | 1.07 (1.06 - 1.07) | 1.22 (1.21 - 1.24) | 1.25 (1.23 - 1.27) |
|  | Moderate-severe | 1.05 (1.04 - 1.06) | 1.17 (1.15 - 1.19) | 1.18 (1.16 - 1.20) |
|  |  |  |  |  |
| **Out-of hour contacts, adjusted** | | |  |  |
|  |  | Mother |  |  |
|  | Mental health condition | No mental health condition | Minor | Moderate-severe |
| Father | No mental health condition | Reference | 1.26 (1.25 - 1.28) | 1.39 (1.37 - 1.40) |
|  | Minor | 1.15 (1.13 - 1.17) | 1.37 (1.34 - 1.41) | 1.49 (1.44 - 1.54) |
|  | Moderate-severe | 1.20 (1.17 - 1.22) | 1.35 (1.30 - 1.40) | 1.43 (1.39 - 1.48) |
|  |  |  |  |  |
| **ER contacts, adjusted** | | |  |  |
|  |  | Mother |  |  |
|  | Mental health condition | No mental health condition | Minor | Moderate-severe |
| Father | No mental health condition | Reference | 1.15 (1.11 - 1.18) | 1.37 (1.32 - 1.42) |
|  | Minor | 1.15 (1.10 - 1.19) | 1.25 (1.16 - 1.33) | 1.39 (1.28 - 1.53) |
|  | Moderate-severe | 1.33 (1.26 - 1.40) | 1.27 (1.15 - 1.41) | 1.37 (1.25 - 1.49) |
|  |  |  |  |  |
| **Inpatient contacts, adjusted** | | |  |  |
|  |  | Mother |  |  |
|  | Mental health condition | No mental health condition | Minor | Moderate-severe |
| Father | No mental health condition | Reference | 1.26 (1.25 - 1.28) | 1.39 (1.37 - 1.41) |
|  | Minor | 1.11 (1.09 - 1.13) | 1.32 (1.29 - 1.37) | 1.47 (1.41 - 1.53) |
|  | Moderate-severe | 1.12 (1.10 - 1.15) | 1.27 (1.22 - 1.34) | 1.41 (1.35 - 1.46) |
|  |  |  |  |  |
| **Outpatient contacts, adjusted** | | |  |  |
|  |  | Mother |  |  |
|  | Mental health condition | No mental health condition | Minor | Moderate-severe |
| Father | No mental health condition | Reference | 1.23 (1.20 - 1.26) | 1.31 (1.27 - 1.35) |
|  | Minor | 1.08 (1.05 - 1.12) | 1.26 (1.19 - 1.33) | 1.55 (1.43 - 1.68) |
|  | Moderate-severe | 1.10 (1.05- 1.15) | 1.20 (1.10 - 1.32) | 1.49 (1.37 - 1.61) |
