# Supplementary figures and images for: Parental mental health conditions and use of healthcare services in children the first year of life– a register-based, nationwide study
Source: BMC Public Health. 2021 Mar 21;21:557. doi: 10.1186/s12889-021-10625-y (PMC7981963; doi:10.1186/s12889-021-10625-y)

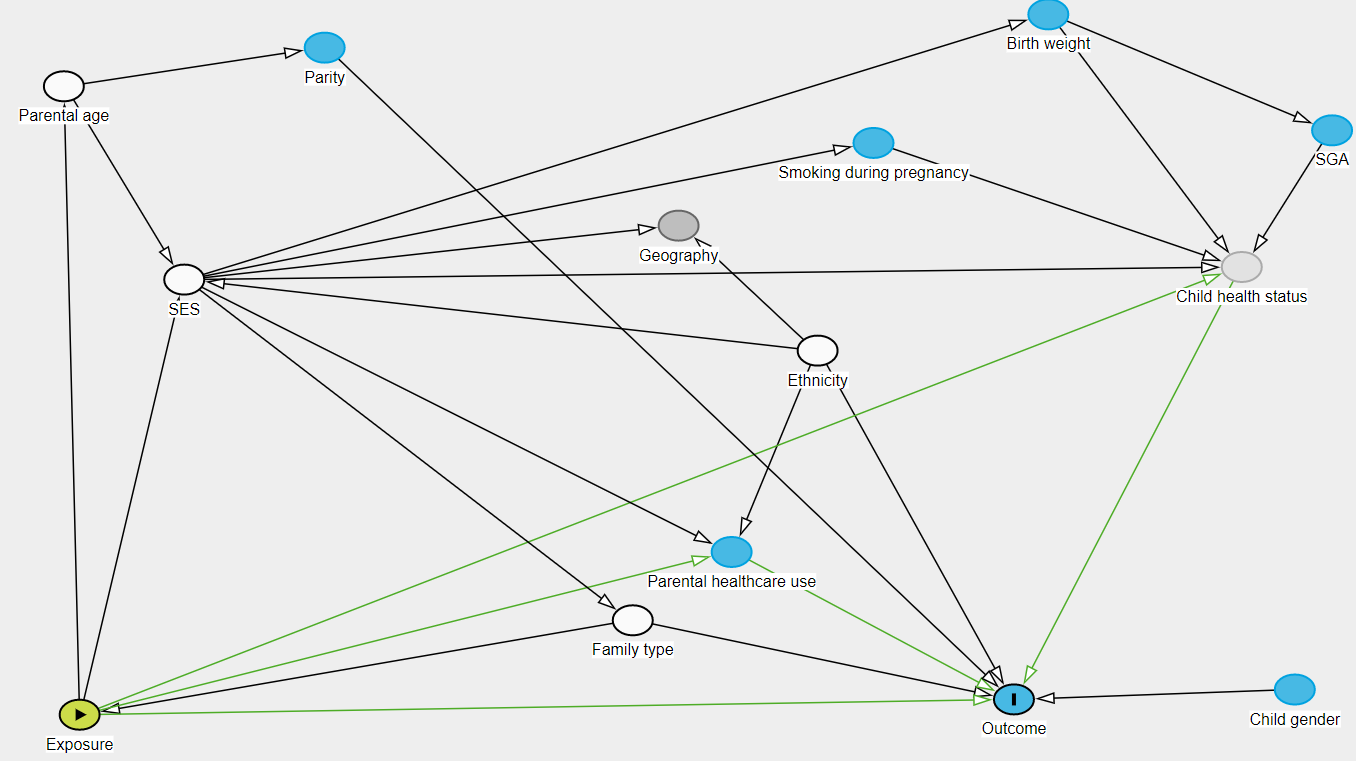

Supplement: Supplementary file 7 — Additional file 7: Supplementary figure 1. Directed acyclic graph. [file 12889_2021_10625_MOESM7_ESM.png]
